# Prospective Study on Self-Calibrating Continuous Glucose Monitoring Practicality and Accuracy in Noncritically Ill COVID-19 Hospitalized Patients

**DOI:** 10.1155/jdr/7538573

**Published:** 2025-08-21

**Authors:** Choompunuj Sakjirapapong, Sirinart Sirinvaravong, Lukana Preechasuk, Nuntakorn Thongtang

**Affiliations:** ^1^Department of Medicine, Mahidol University Faculty of Medicine Siriraj Hospital, Bangkok, Thailand; ^2^Diabetes, Thyroid and Endocrine Center, Phyathai2 International Hospital, Bangkok, Thailand; ^3^Siriraj Diabetes Center of Excellence, Mahidol University Faculty of Medicine Siriraj Hospital, Bangkok, Thailand

**Keywords:** accuracy, continuous glucose monitoring, COVID-19

## Abstract

**Aims:** This study is aimed at evaluating the accuracy and feasibility of real-time continuous glucose monitoring (rt-CGM) in non-ICU hospitalized adult COVID-19 patients who had hyperglycemia requiring insulin therapy during admission.

**Materials and Methods:** Medtronic Guardian Sensor 3 and transmitter were placed on the patient's abdomen. The patient performed a self-calibration of CGM via the application installed in the smartphone. Paired CBG and sensor glucose values were analyzed for accuracy of CGM using mean absolute relative difference (MARD) and Clarke error grid analysis (CEGA).

**Results:** Fifteen patients were enrolled. Mean age was 48.6 ± 17.9 years; 53.3% were female. Thirteen patients (86.7%) had pre-existing diabetes. Mean HbA1c was 10.6 ± 3.6%. Mean duration of CGM use was 6 ± 1.2 days, and mean calibration was 2.6 ± 0.7 times/day. There were 253 paired CBG and CGM measurements. The overall MARD was 9.9 ± 9.3%. The lowest MARD was observed in the CBG range of 70–180 mg/dL (9.6 ± 9.0%) and on Day 3 of sensor wear (8.0 ± 4.6%). The percentages of glucose readings within 15/15%, 20/20%, and 30/30% were 81.0%, 89.7%, and 95.3%, respectively. A total of 99.2% of the data points were in Zones A and B of CEGA, and none were in Zone E. CGM reduced POC testing by approximately 30%.

**Conclusions:** Rt-CGM use in hospitalized patients with COVID-19 infection demonstrates high accuracy, reduces the frequency of CBG testing, and preserves medical resources. The patient's self-calibration of rt-CGM in this setting is feasible. Although the COVID-19 pandemic tends to improve, our research could be applied to new emerging infectious diseases.

**Trial Registration:** Thai Clinical Trials Registry: TCTR20230426007.

## 1. Introduction

Dysglycemia in patients with or without diabetes mellitus increases the risk of in-hospital complications, particularly infection and prolonged length of stay [1]. Among patients with COVID-19 infection, those with diabetes have a higher disease severity and are at a greater risk of ICU admission, COVID-19-related complications, and mortality than those without [2–4]. Optimal glycemic control is associated with favorable clinical outcomes in COVID-19 hospitalized patients. In the retrospective study of patients with pre-existing diabetes who had COVID-19 infection, well-controlled blood glucose during hospitalization was associated with markedly reduced mortality [5].

Management of hyperglycemia in hospitalized patients usually requires insulin therapy, especially in those with pre-existing diabetes, and a close monitoring of glucose levels is essential. Due to the scarcity of personal protective equipment (PPE) and the risk of contracting infection among healthcare workers during the COVID-19 pandemic, routine frequent capillary blood glucose (CBG) assessment was difficult to perform. Consequently, in April 2020, the US Food and Drug Administration (FDA) approved the use of continuous glucose monitors (CGMs) in patients with diabetes who were hospitalized due to COVID-19 infection [6].

The 2020 CGMs and Automated Insulin Dosing Systems in the Hospital Consensus Guideline recommended prescribing CGMs to reduce the frequency of nurse contact for glucose testing and the PPE usage for patients with highly contagious infectious diseases who were in isolation [7]. According to previous studies on the accuracy of CGMs, mean absolute relative difference (MARD) was 9.77% in non-ICU patients using Dexcom G6 [8] and 11.1%–13.1% in critically ill adult COVID-19 patients using Medtronic Guardian Connect or Dexcom G6 [9]. However, there have been no clinical trials using Medtronic Guardian Connect real-time continuous glucose monitoring (rt-CGM) in non-ICU hospitalized adult COVID-19 patients.

The aim of this study was to evaluate the accuracy and feasibility of Medtronic Guardian Connect rt-CGM in non-ICU hospitalized adult COVID-19 patients.

## 2. Materials and Methods

### 2.1. Study Design and Study Population

This is a single-center prospective study of non-ICU hospitalized adult patients with COVID-19 infection. Inclusion criteria were hospitalized patients ≥ 18 years old with a positive COVID-19 RT-PCR test who had hyperglycemia requiring insulin therapy during admission in non-ICU units and the capability to use rt-CGMs and perform self-monitoring of CBG. Exclusion criteria included coagulopathy (INR over 3), thrombocytopenia (platelet count less than 20,000/uL), hypotension (defined as BP less than 90/60 mmHg) or poor tissue perfusion, multiorgan failure, significant edema, current treatment with dual antiplatelet therapy or medications known to interfere with the accuracy of CGMs such as hydroxyurea, intravenous ascorbic acid, and paracetamol more than 4 g in 24 h.

### 2.2. Study Protocol

We enrolled the eligible research participants from non-ICU COVID-19 wards at Siriraj hospital, Thailand, between August 2021 and January 2022. All patients provided written informed consent upon enrollment. The study's protocol was approved by the Siriraj Institutional Review Board (SIRB) (COA No. 644/2021). Due to the COVID-19 pandemic emergency situation, we urgently conducted our clinical trial to assist glucose control in hospitalized patients. Along with this study, we concurrently conducted another study in non-COVID-19 infection, Type 2 diabetes hospitalized patients to assess the accuracy and feasibility of rt-CGM [10].

After the recruitment, we collected demographic information, type and previous treatment of diabetes (if any), comorbidities, respiratory support status, and severity of COVID-19 infection by Ordinal Scale for Clinical Improvement of the World Health Organization (WHO) [11] and National Early Warning Score (NEWS)2 [12]. Data on COVID-19 treatment such as type, dose, and duration of medication, including use of glucocorticoid treatment, were also recorded.

The Guardian Connect rt-CGM system (Medtronic Inc., Northridge, California, United States) was used in this study. Prior to the insertion of the device, patients were trained to perform CBG testing using StatStrip and StatStrip Xpress glucose meter (Nova Biomedical, Cheshire, United Kingdom) and self-calibration of CGM via the Guardian Connect application on a smartphone. Then, a trained researcher placed a 7-day Medtronic Guardian Sensor 3 and transmitter on the participant's abdomen. Calibration was required three times per day on the first day and then at least twice a day (in the morning and evening) on the following days (according to the manufacturer's recommendation). The CGM system automatically received sensor glucose (SG) values every 5 min and displayed them on the application.

During the study period, the physicians were able to monitor the rt-CGM data of the patients from the Medtronic CareLink website. Diabetic medications were adjusted according to the attending physician's discretion. If there were low (< 70 mg/dL) or high (> 250 mg/dL) glucose level alarms, the patients were alerted to confirm their CBG, and the treatment decision was made according to the CBG level. The sensor and transmitter were removed after 7 days of attachment or when the patients were discharged from the hospital. CBG and SG values, diabetic medication, and adverse events were recorded during the trial period.

### 2.3. Statistical Analysis

The sample size was calculated based on estimation of the mean formula. According to the previous study [9], overall MARD was 13.1% and the standard deviation (SD) of MARD was 10%; a sample size of 15 would provide a precision error of 5% and a 95% confidence interval.

The categorical variables were presented in percentages. The continuous variables were presented as mean and SD if normally distributed and as median and interquartile range (IQR) if not normally distributed.

Paired CBG and SG values were analyzed for accuracy of CGM using bias, MARD and Clarke error grid analysis (CEGA) [13]. SG values used in the calculation were the values closest in time to CBG readings. Incorrect or false calibration (defined as more than 10-min delay in calibration or repeated calibration using the same CBG value more than twice) was excluded from the analysis. MARD was calculated as the average relative difference between paired SG and CBG values and expressed as a percentage [14]. The CGM performance analysis also included median absolute relative difference (median ARD), and the proportion of SG values within 15 mg/dL if CBG values were less than 70 mg/dL or 15% if CBG values were ≥ 70 mg/dL (± 15/15%). The same measurement for ± 20/20%, ± 30/30%, and ± 40/40% criteria was also calculated. A paired *T*-test was used to compare time in range (TIR), time below range (TBR), and time above range (TAR) of Day 1 and the last 72 h of sensor wear. *p* values of less than 0.05 were considered statistically significant. The statistical analysis was conducted using IBM SPSS Statistics Version 20.

We also estimated the percent reduction of the frequency of CBG testings while using CGM. The frequency of CBG testing used in the calculation followed the standard of care guidelines specific to each insulin regimen. For individuals on a basal-bolus insulin regimen, CBG should be tested a minimum of four times daily. For those using premixed insulin or basal-only insulin regimens, testing should occur at least twice daily and once daily, respectively [15].

## 3. Results

### 3.1. Participant Characteristics

Seventeen participants were enrolled. Two were excluded after 1 day of CGM insertion (one participant had dislodgement of the sensor and the other had repeatedly entered wrong CBG values for calibration thrice, leading to sensor error). The baseline characteristics of 15 participants are described in Table 1.

Mean age of the participants was 48.6 ± 17.9 years, and 53% were female. The three most common comorbidities were hypertension, dyslipidemia, and cerebrovascular disease. The majority of participants had pre-existing Type 2 diabetes, whereas 13.3% of them had newly diagnosed diabetes of uncertain type, for which steroid-induced hyperglycemia or pre-existing Type 2 diabetes was suspected. None of the participants had hyperglycemic emergency within 2 weeks before enrollment. Mean HbA1c was 10.6 ± 3.6% and median total daily insulin dose before enrollment was 0.4 (IQR 0.3–0.8) unit per kilogram of body weight. NEWS 2 score was used to assess the severity of COVID-19 infection; 66.7% had low score (≤ 4) indicating mild disease, while 33.3% had more severe disease (Table 1).

Mean duration of CGM use was 6 ± 1.2 days and mean frequency of calibration was 2.6 ± 0.7 times per day. The maximum frequency of calibration was four times a day. Incorrect or false calibration occurred in 2.8% of all calibrations. None of these participants had adverse events from CGM insertion or CGM wear.

### 3.2. MARD and Bias

There were 253 paired SG and CBG measurements. The overall MARD was 9.9% ± 9.3% and the median ARD was 7.7% (IQR 3.6, 13.1). The lowest MARD was observed in the CBG range of 70–180 mg/dL (9.6 ± 9.0%), as shown in Tables 2 and 3. We also analyzed the MARD values on each day of sensor wear; the lowest MARD was observed on Day 3 of sensor wear (8.0 ± 4.6%). The Bland–Altman analysis comparing SG and CBG had a bias of −15.5 mg/dL with 95% limits of agreement from −64.3 and +33.3 mg/dL.

### 3.3. Agreement Analysis

Table 4 shows the agreement between paired sensor and CBG values. The overall agreement rate (20%) was 89.7% (95% CI 85.3, 93.2). The agreement was then analyzed by different glucose ranges; 80.4% of SG readings were within 15% agreement with CBG in the CBG range of 70–180 mg/dL and 81.0% in the CBG range of > 180 mg/dL.

### 3.4. CEGA

**Figure 1:**
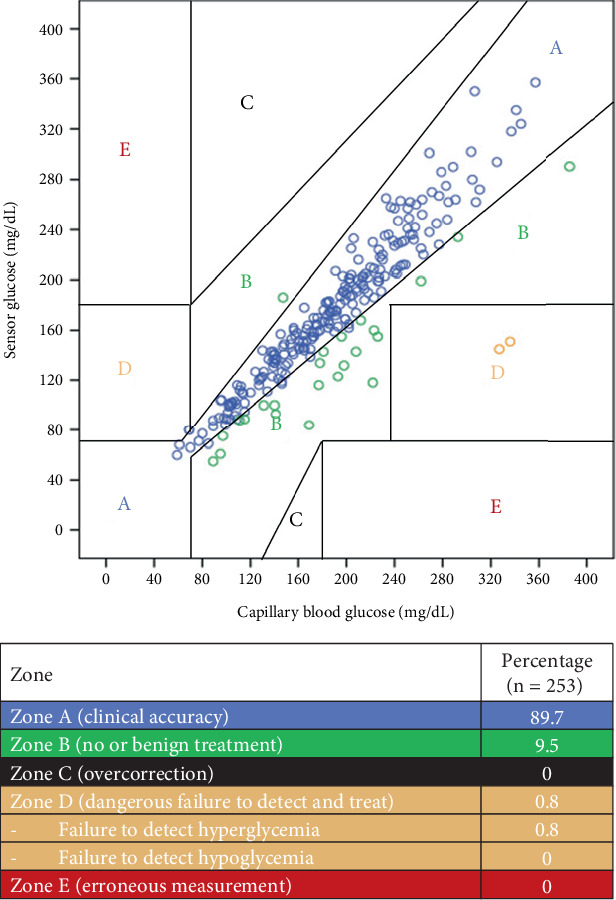
shows Clarke error grid analysis of paired SG-CBG values. The *X* axis of this graph represents CBG values, and the *Y* axis represents sensor glucose values.

The paired SG and CBG values were stratified into 5 zones, as shown in Figure 1. Zone A represents SG values that deviate from the CBG by not more than 20% or in the same hypoglycemic range (less than 70 mg/dL) of both SG and CBG. Zone B represents SG values that deviate from the CBG by more than 20% but would lead to benign or no treatment. A total of 99.2% of the data points were in Zones A and B, which were clinically acceptable. There was no failure to detect hypoglycemia and no erroneous measurement in our study.

### 3.5. CGM-Derived Parameters

Overall median TIR, TAR, and TBR were 59.0% (IQR 49.0–73.0), 39.0% (IQR 26.5–51.0), and 0.0% (IQR 0.0–1.5), respectively. Percent TIR, TBR, and TAR on each day of sensor wear were analyzed. Percent TIR on Day 1 was 57.9 ± 22.9 and increased to 64.9 ± 18.1 in the last 72 h of sensor wear (Table 5). However, the change was not statistically significant (*p* = 0.39).

### 3.6. Reduction of Point-of-Care (POC) Blood Glucose (POC-BG) Testing

POC blood glucose testing was reduced by approximately 33% on the first 3 days and 30% in total during the entire sensor wear time compared with the predicted numbers of POC glucose testing based on the traditional care of approximately 2–4 times per day.

## 4. Discussion

Since the US FDA granted nonobjection to the use of CGM in hospitalized patients in response to the COVID-19 pandemic, there have been only a few clinical trials on the performance and safety of CGM in noncritically ill diabetic patients with COVID-19 infection. We did a study to investigate the accuracy and feasibility of Medtronic Guardian Connect rt-CGM in non-ICU hospitalized adult COVID-19 patients. Our study showed that Medtronic Guardian Connect rt-CGM had a high accuracy with the overall MARD of 9.9 ± 9.3%. The acceptable MARD varies among different clinical settings, ranging from less than 10% to 14% [16]. Compared to the study of Tingsarat et al., which used the same sensor model, their MARD was 6.6%, which was lower than ours [17]. However, their study was performed in critically ill non-COVID-19 patients, and it is worthwhile to note that the calibration was performed by nursing staff, whereas in our study, calibration was carried out by the patients.

Our results were concordant with another previous study that evaluated the accuracy of Medtronic rt-CGM in COVID-19 patients. Sadhu et al. reported a MARD of 13% in ICU hospitalized patients using the Medtronic guardian sensor, and POC test reduction was 30% [9]. Other previous studies of the accuracy of Dexcom G6 CGM in COVID-19 hospitalized patients showed the MARDs range between 9.8% and 14.8%, which was comparable to the MARD in our study [8, 18, 19].

The agreement between paired SG and CBG values from our study showed that the overall agreement rate (20%) was 89.7%. Our result fitted into the acceptable range defined in the US FDA measurement accuracy criteria of integrated CGM system [20]. However, this measurement accuracy system was meant for personal ambulatory usage, and to date, there has been no international consensus on the accuracy standard for inpatient CGM use.

Furthermore, we analyzed CEGA to evaluate the clinical accuracy of CGM. Ninety-nine percent of the data points from our study were in Zones A and B of CEGA. Our result was similar to the studies of Tingsarat et al. [17] and Sadhu et al. [9]. Their studies showed that 99.7% and 100% of the data points were in Zones A and B, respectively, whereas the results from the studies of Dexcom G6 CGM ranged between 98.0% and 99.6% [8, 18, 19].

Dysglycemia in patients with COVID-19 infection is associated with worse clinical outcomes. However, achieving good glycemic control requires frequent blood glucose monitoring. The 2022 *Management of Hyperglycemia in Hospitalized Adult Patients in Non-Critical Care Settings* guideline by the Endocrine Society recommends the use of rt-CGM in conjunction with confirmatory bedside POC-BG testing in insulin-treated diabetes patients admitted with noncritical illness [21]. Although newer models of rt-CGM do not require calibration, many international guidelines still advise the use of POC-BG testing alongside CGM [22].

Glucose monitoring in hospitalized COVID-19-infected patients using traditional fingerstick POC glucose testing is often less frequent than the usual standard of care. Wearing rt-CGM in COVID-19 hospitalized patients led to a reduction in the number of POC glucose tests by 30% and PPE use for entering the isolation unit in our study. The 2022 Management of Individuals With Diabetes at High Risk for Hypoglycemia Endocrine Society Clinical Practice Guideline also suggests the CGM use for particular inpatients at high risk for hypoglycemia, including patients with contagious diseases (e.g., COVID-19) to reduce exposure of healthcare personnel and PPE usage [23]. However, the US FDA does not currently approve the initiation of CGM in the inpatient setting other than for COVID-19 infection. Future studies will be needed to evaluate the accuracy of CGM use apart from the COVID-19 situation. Although the COVID-19 pandemic has subsided, our results can be applied to the glycemic management of other emerging contagious diseases.

The strength of our study included that this was the first clinical trial of rt-CGM in hospitalized adult patients that allowed the patients to perform self-calibration, which further reduced the contact time of healthcare personnel with patients who had a communicable disease. We also showed percent TIR, TAR, and TBR on each day of sensor wear, which had never been assessed in the previous studies. Although the trend of TIR improvement was not statistically significant, our findings could be used to guide the future directions of CGM research.

There were several limitations in our study. First, our sample size was relatively small. Second, the comparison data points on Days 5–7 were limited because most of the patients were discharged before the end of a 1-week period. Finally, we did not investigate the patients' and healthcare provider team's satisfaction. However, a study of the feasibility of Medtronic Guardian Connect in the inpatient diabetes unit by Dillmann et al. reported that 94% of the patients and nurses found that rt-CGM use was useful [24].

## 5. Conclusions

Rt-CGM use in hospitalized patients with COVID-19 infection demonstrates high accuracy and reduces the frequency of CBG testing. The patient's self-calibration of rt-CGM in this setting is feasible. Although the CGM pandemic tends to improve, our research could apply to new emerging infectious diseases.

## Figures and Tables

**Table 1:** Baseline characteristics of the study patients.

**Baseline characteristics**	**N** = 15
Age—year (mean ± SD)	48.6 ± 17.9
Female sex—no. (%)	8 (53.3)
Race—no. (%)	
Thai	13 (86.7)
Lao	2 (12.3)
BMI—kg/m^2^ (Mean ± SD)	27.5 ± 5.6
Comorbidities—no. (%)	
Hypertension	2 (13.3)
Dyslipidemia	1 (6.7)
Cerebrovascular disease	1 (6.7)
Coronary artery disease	0 (0.0)
Diabetes mellitus—no. (%)	
Pre-existing T2DM	10 (66.7)
Pre-existing T1DM	3 (20)
Newly diagnosed DM	2 (13.3)
Diabetes medication—no. (%)	
No medication	7 (46.7)
OHA^†^	4 (26.7)
Insulin	4 (26.7)
HbA1c—%	10.6 ± 3.6
Median eGFR—mL/min/1.73 m^2^ (IQR)	106.2 (77.5–123.2)
Steroid use—no. (%)	9 (60)
Median equivalent prednisolone dose per day (min–max) (mg/day)	50 (25–75)
Total daily insulin dose before enrollment	
Median value—unit (IQR)	28 (18–49)
Median unit per kg (IQR)	0.4 (0.3–0.8)
Respiratory support—no. (%)	
No respiratory support	7 (46.7)
O_2_ cannula	5 (33.3)
O_2_ mask	2 (13.3)
HFNC^‡^	1 (6.7)
Organ failure—no. (%)	
No organ failure	14 (93.3)
Renal failure	1 (6.7)
National Early warning score (NEWS)—no. (%)	
0–4	10 (66.7)
5–6	2 (13.3)
≥ 7	3 (20.0)

^†^Oral hypoglycemic agent.

^‡^High-flow nasal cannula.

**Table 2:** Mean absolute relative difference (MARD) value. MARD of the different CBG ranges.

**Index**	**Capillary blood glucose range (mg/dL)**			**Total**
	**< 70**	**70–180**	**> 180**	
Paired SG and CBG readings	4	112	137	253
Mean ARD ± SD (%)	11.3 ± 6.7	9.6 ± 9.0	10.2 ± 9.6	9.9 ± 9.3
Median ARD (Q1, Q3) (%)	13.7 (6.6, 15.9)	7.1 (3.5, 11.5)	7.9 (4.0, 13.7)	7.7 (3.6, 13.1)
95% CI	0.6, 22.0	7.9, 11.3	8.6, 11.8	8.8, 11.1

**Table 3:** Mean absolute relative difference (MARD) value. MARD on each day of sensor wear.

**Day**	**Number of paired SG and CBG readings**	**Mean ** **A** **R** **D** ± **SD**	**Median ARD (Q1, Q3)**	**95% CI**
Day 1	69	11.0 ± 12.1	7.3 (2.6, 15.1)	8.1, 13.9
Day 2	45	8.8 ± 8.6	7.4 (3.4, 12.5)	6.2, 11.3
Day 3	45	8.0 ± 4.6	7.5 (4.4, 11.5)	6.6, 9.4
Day 4	39	9.6 ± 8.1	7.9 (3.5, 12.7)	7.0, 12.2
Day 5	27	10.4 ± 8.8	6.3 (3.9, 14.2)	6.9, 13.8
Day 6	20	11.3 ± 8.8	8.5 (5.3,13.9)	7.1,15.4
Day 7	8	15.2 ± 12.9	12.0 (5.4, 24.1)	4.3, 26.0
Total	253	9.9 ± 9.3	7.7 (3.6, 13.1)	8.8, 11.1

**Table 4:** Agreement between paired SG-CBG values in the range of CBG levels.

**Agreement level**	**± 10/10% (mg/dL)**	**± 15/15% (mg/dL)**	**± 20/20% (mg/dL)**	**± 30/30% (mg/dL)**	**± 40/40% (mg/dL)**
Total	63.6%	81.0%	89.7%	95.3%	98.4%
95% CI	57.4, 69.6	75.7, 85.7	85.3, 93.2	91.9, 97.8	96.0, 99.6
< 70 mg/dL	50%	100%	100%	100%	100%
95% CI	6.8, 93.2	NA^†^	NA^†^	NA^†^	NA^†^
70–180 mg/dL	66.1%	80.4%	89.3%	95.5%	99.1%
95% CI	56.5, 74.8	71.8, 87.3	82.0, 94.3	89.9, 98.5	95.1, 99.9
> 180 mg/dL	62.0%	81.0%	89.8%	94.9%	97.8%
95% CI	53.4, 70.2	73.4, 87.2	83.5, 94.3	99.8, 97.9	93.7, 99.6

^†^Not available.

**Table 5:** Percent TBR, TIR, and TAR on each day of sensor wear.

**Day**	**N**	**%TBR** ^**†**^	**%TIR** ^**‡**^	**%TAR** ^**§**^
1	15	0.9 ± 3.6	57.9 ± 22.9	41.1 ± 24.0
2	15	0.9 ± 2.2	54.4 ± 23.7	44.1 ± 24.5
3	15	0.6 ± 1.2	54.5 ± 22.4	44.9 ± 23.2
4	15	0.8 ± 2.3	58.9 ± 21.5	40.3 ± 22.6
5	11	0.1 ± 0.3	59.2 ± 25.1	40.7 ± 25.3
6	6	0.7 ± 0.8	57.8 ± 19.2	41.5 ± 19.2
7	4	4.3 ± 4.7	64.8 ± 19.9	31.0 ± 23.8
Last 72 h	15	1.3 ± 2.3	64.9 ± 18.1	33.9 ± 19.1
Total	15	0.9 ± 2.4	58.5 ± 21.5	40.5 ± 22.4

*Note:* Data was reported as mean ± SD.

^†^Time below range.

^‡^Time in range.

^§^Time above range.

## Data Availability

The data that support the findings of this study are available from the corresponding author upon reasonable request.
